# Machine learning outcome prediction using stress perfusion cardiac magnetic resonance reports and natural language processing of electronic health records

**DOI:** 10.1016/j.imu.2023.101418

**Published:** 2024

**Authors:** Ebraham Alskaf, Simon M. Frey, Cian M. Scannell, Avan Suinesiaputra, Dijana Vilic, Vlad Dinu, Pier Giorgio Masci, Divaka Perera, Alistair Young, Amedeo Chiribiri

**Affiliations:** a School of Biomedical Engineering & Imaging Sciences, King’s College London, United Kingdom; b Department of Cardiology, University Hospital Basel, Basel, Switzerland; c Department of Biomedical Engineering, Eindhoven University of Technology, Eindhoven, Netherlands; d King’s College London, United Kingdom

**Keywords:** Machine learning, Coronary artery disease, Cardiac magnetic resonance, Electronic health records, Outcome prediction, Natural language processing

## 1 Introduction

Stress perfusion cardiac magnetic resonance (SP-CMR) imaging is a guideline-backed clinical test performed in patients with known or suspected coronary artery disease (CAD). This technique allows to test for inducible perfusion defects in the myocardium of the left ventricle, which allows to judge on the presence and location of haemodynamically significant coronary artery stenoses [1], and to assess for microvascular ischaemia [2]. SP-CMR is increasingly used in cardiac imaging and has been well validated against other imaging modalities such as invasive coronary angiography or fractional flow reserve (FFR) assessment [3,4]. CE-MARC study [5] and several large-scale studies have shown non-inferiority or superiority compared to Single Photon Emission Computed Tomography (SPECT) stress perfusion imaging in prediction of major adverse cardiovascular events (MACE), hence SP-CMR is increasingly becoming an established diagnostic tool for CAD and a marker of prognosis [6]. It was shown in the ISCHAEMIA trial that an initial invasive strategy as compared to an initial conservative strategy did not reduce the risk of ischaemic cardiovascular events or mortality in patients with stable CAD and moderate or severe ischaemia over a median follow up of 3.2 years [7], and the MR-INFORM study showed that SP-CMR was associated with a lower incidence of coronary revascularisation than FFR and was non-inferior to FFR with respect to major adverse cardiac events, amongst patients with stable angina and risk factors for coronary artery disease [8]. This highlights the importance of SP-CMR as an accurate non-invasive assessment tool for risk stratification and management planning of patients with known or suspected CAD, in addition to the assessment of ventricular function, myocardial scar and viability all in one exam. It was given class I recommendation in the European Society of Cardiology (ESC) guidelines 2019 [9] and the American College of Cardiology (ACC) guidelines 2021 [10].

In the majority of health care systems, data from medical records are stored in unstructured formats. Except for investing significant resources to collect and restructure this data, it cannot be accessed and used for quality audits or scientific purposes. Unstructured data is growing far faster than structured data, so it is imperative to develop methods to extract information from it. Artificial intelligence (AI) is an emerging technology capable of extracting and processing unstructured text using natural language processing (NLP) algorithms to recognise patterns in the text and relate these to structured information. Furthermore, machine learning (ML) methods are also well suited to predict clinical outcomes accurately, this can be performed using non-linear modelling that learn the outcomes associated with standardised input data and produce accurate outcome predictions when applied to new data [11].

The objectives of this study are: (1) to test whether an AI-based tool using NLP can extract structured information from large unstructured electronic health records (EHR) data; (2) to examine the prognostic value of stress CMR retrospectively; and (3) to apply ML techniques for outcome prediction based on the extracted SP-CMR reports and clinical variables.

## 2 Methods

### 2.1 Study selection and study design

Study ethical approval was obtained under the Research Ethics Committee reference number: 20/ES/0005. All SP-CMR examinations performed in one centre (Guy’s and St Thomas’ Trust in London, United Kingdom) between April 2011 and March 2021 were identified using the logbook from the administration office and analysed retrospectively. Two different CMR report types were available: an unstructured type between 2011 and 2015, and a structured type initiated in 2016. The list of cases was saved on a text file and contained patients’ names and dates of the studies, these cases were cross-referenced to CMR image archive, cases with no studies identified on archive were excluded.

We included only patients with complete adenosine stress perfusion study and good quality images, who had complete reports. Exclusion criteria were: reports which were blinded for research purposes, conflicting reports description between main body text and summary findings, lack of documented American Heart Association (AHA) segmentation, terminated stress study due to complications, reports with missing tissue characterisation information, contraindications to using stress agent, mass perfusion studies, dobutamine stress studies, lung perfusion studies, poor response to stress agent, and mis-labelled reports which were originally highlighted as perfusion studies but after inspection found to be otherwise.

### 2.2 Data extraction

Data extraction was mainly performed with Cogstack [12], which is a healthcare application framework allows the extraction of information from unstructured data sources, such as EHR in which the majority of the information contents is locked-up (i.e. not programmatically queryable) in multiple formats of unstructured data (i.e. binary words docs, PDFs, images, text fields etc). Once extracted, harmonised and processed, multiple uses of this unstructured data become possible. These include NLP, enterprise search, alerting and cohort selection. These tools allow clinical text to be searched for specific terms using simple or complex syntax, rapidly retrieving the data needed to answer complex queries.

Cogstack is a software for text-based data extraction, and was the main application used to extract patients’ EHR data for this study. The text file containing all patients’ names on record was converted into an Excel spreadsheet and uploaded to an Application Programming Interface (API) written in Python Jupyter Notebook programming language. This returned a list of unique values with their identification (ID) numbers and a list of names. A manual check on all cases was performed against CMR image archive to identify the correct names and IDs. The final list of the names found was then sent to Cogstack to extract baseline characteristics using standard Elasticsearch query and using Python API scripts.

### 2.3 NLP network building

Clinical information was extracted using CogStack from structured fields (demographics) and unstructured free text (clinical notes, discharge summaries, etc). Using medical terms based on Systematic Nomenclature of Medicine Clinical Terms (SNOMED CT), samples of documents were ingested into the Medical Concept Annotation Tool (MedCAT), which is used to link EHRs to biomedical ontologies such as SNOMED-CT and Unified Medical Language System (UMLS) and train NLP models. For this study, SNOMED-CT UK version was used for annotation. Text files were tokenized, lemmatised and pre-processed, then used as inputs into the network with the corresponding labels. Initial self-supervised model was trained using named-entity recognition + linking (NER + L) annotation, this algorithm is used to extract and locate name entities in unstructured text into a pre-defined categories for labelling before training the model. Fine tuning was achieved with supervised learning after a group of expert clinicians labelled a sample of reports with the relevant medical terminology. MedCAT trainer used multiple neural networks architecture (long-short-term-memory (LSTM), gated recurrent unit (GRU), and transformers), and the best performing model was deployed into CogStack. Full details on models building of our organisation has been published [13].

**Fig. 1:**
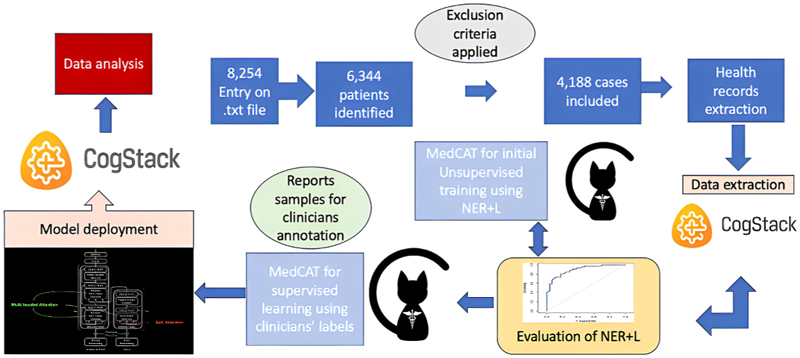
Diagram shows the data extraction process using Cogstack application and training NLP model. NER + L; named-entity recognition + linking, LP; natural language processing.

Cardiovascular risk factors were: smoking, hypertension (HTN), chronic kidney disease (CKD), diabetes mellitus (DM), dyslipidaemia, history of heart failure (HF), cerebrovascular event (CVA) and myocardial infarction (MI). Arrythmias included: atrial fibrillation (AF) and flutter, ventricular tachycardia (VT), ventricular fibrillation (VF), first, second, and third degree heart blocks. The CMR variables were extracted manually as data ingestion into MedCAT was not possible at the time of writing this manuscript, these included: left ventricular ejection fraction (LVEF), right ventricular ejection fraction (RVEF), field strength, stress agents, binary stress perfusion AHA segments, and binary AHA late gadolinium enhancement (LGE) segments. All data extracted following this process were analysed. The data extraction process is depicted in Fig. 1.

### 2.4 Data analysis

#### 2.4.1 Statistical analysis

Categorical variables were expressed using number and percentage, continuous variables were expressed using mean and standard deviation.

The population was divided into three age subgroups (<65 years, 65–75 years, >75 years). The difference in baseline characteristics, risk factors and CMR data between subgroups was tested using Kruskal Wallis test for categorical variables and One Way ANOVA for continuous variables.

The best survival model was determined using Akaike’s Information Criterion (AIC) aiming for the model with the lowest AIC. Survival for all-cause mortality was assessed by Log Normal curves and difference among the group tested using the Log-rank test. Follow up was calculated as the mean time of CMR scan date to event date, and all cases with shorter duration were right censored.

Multivariate analysis of survival was tested using Cox proportional hazard model to construct two models: (1) clinical model (model-1) including age, gender, HF, DM, HTN, dyslipidaemia, CKD, smoking history and CVA, and (2) CMR model (model-2) including stress perfusion and LGE. Survival analysis for patients with positive ischaemia or LGE from CMR model were also tested for individual coronary territories outcome.

Multivariate analysis of ventricular arrhythmia was constructed using multivariate regression analysis and the interaction between clinical and CMR variables was tested using heatmap correlation.

A separate survival analysis was performed on CMR continuous variables from LVEF and RVEF using incremental values with the application of Accelerated Failure Time (AFT) applied on Weibull survival method.

#### 2.4.2 Machine learning development

Data were split into 80 % for training and 20 % for testing with K-fold cross validation (number of folds = 5). Different machine learning models were trained and tested to predict all-cause mortality, these included: support vector machine (SVM), random forest (RF), extreme gradient boosting (XGBoost) classifier, and ensemble classifier combining SVM, RF and XGBoost using soft voting based on the argmax of the sum of predicted probabilities, which is recommended for an ensemble of well-calibrated classifiers.

All models were compared with multivariate regression (linear prediction) model using area under the curve (AUC), accuracy and F1 score. AUCs were compared using DeLong test. Survival models were also compared before and after the addition of CMR variables to test for their significance.

All statistical analysis was performed using Python programming language, version 3.8.8.

## 3 Results

### 3.1 NLP model performance

The best performing model was bidirectional LSTM neural network. CogStack NLP model performance exceeded 0.82 in precision, recall and F1 scores, indicating high performance level: precision ranged from 0.949 for HTN to 1.0 for AF, recall ranged from 0.825 for DM to 0.959 for HF, F1 score ranged from 0.875 in AF to 0.95 in CKD.

### 3.2 Baseline characteristics

A significant proportion of the cases (26 %, 1620 cases) were excluded from the baseline sample size of 6344 due to mis-labelled CMR stress report as the protocol was standard, lung perfusion, dobutamine stress study, or mass perfusion protocol. A further 12 cases were excluded as reports were not found. Finally, 524 cases (8 %) were excluded due to incomplete stress perfusion studies for various reasons, contraindications to the use of a stress agent (2 %), other technical difficulties (2 %), and adverse events to the use of stress agents (2 %).

**Fig. 2:**
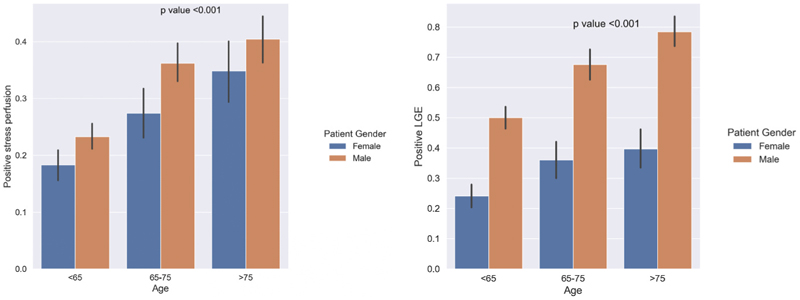
Bar plots showing the proportion of cases with presence of ischaemia (left) and ischaemic LGE (right). LGE; late gadolinium enhancement.

The total number of cases included in the final analysis was 4188 (Fig. 1). Baseline characteristics stratified by age group are shown in Table 1. Mean age was 64 ± 14 years, 63 % were males and 37 % were females. The total number of reported deaths found on record was 252 cases. This was significantly more frequent in older patients (P < 0.001). There was significant difference between age subgroups in risk factors for CAD with more prevalence in patients ≥65 years old, this includes: smoking, HTN, dyslipidaemia, CVA, CKD and HF. There was no significant difference in the incidence of ventricular arrhythmias between age subgroups, but there was a lower incidence of atrial fibrillation and atrial flutter patients younger than 65 years. Finally, positive LGE was more common in patients aged 75 or older than other two groups (Fig. 2).

### 3.3 Survival analysis

Mean follow-up duration was calculated at 1090 days, and total number of cases censored for survival analysis was 1780 (43 %). Log Normal curves showed that median survival was 659 days and ranges from 9 to 3441 days with 95 % confidence interval.

**Fig. 3:**
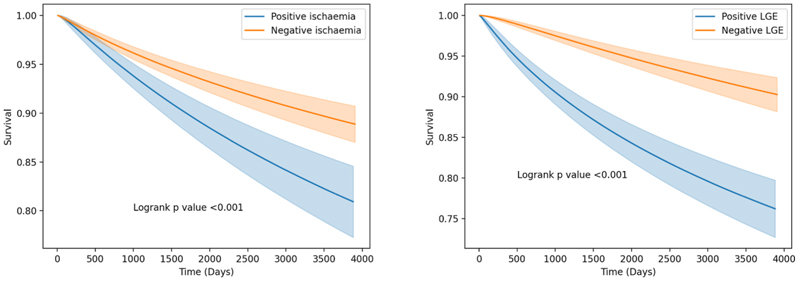
Survival analysis of patients referred for stress perfusion CMR depending on presence of ischaemia (left) and myocardial infarction or ischaemic LGE (right). LGE; late gadolinium enhancement.

Log Normal curve analysis showed lower survival for patients with positive ischaemia on stress perfusion CMR reports (Logrank p < 0.001), and lower survival for patients with positive LGE imaging (Logrank p < 0.001), as shown in Fig. 3.

There was no significant difference in survival based on the ischaemic coronary territory involved (Logrank p = 0.643). In respect to the presence of scar, there was also no significant difference in survival based on the coronary territory involved (Logrank p = 0.305).

### 3.4 Clinical and CMR predictors of all-cause mortality

**Fig. 4:**
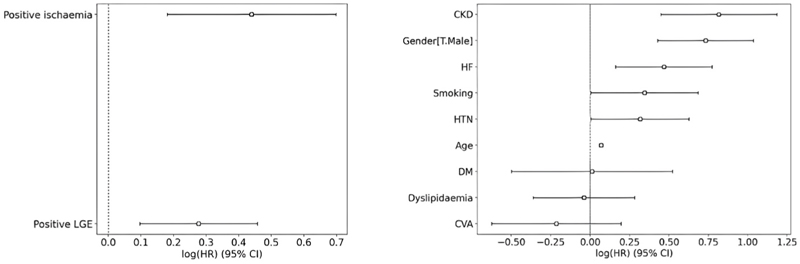
All-cause mortality Cox HR model showing CMR model (left) and clinical model (right). CKD; chronic kidney disease, CVA; cerebrovascular event, DM; diabetes mellitus, HF; heart failure, HTN; hypertension, LGE; late gadolinium enhancement.

The results of multivariate Cox proportional hazard analyses for the two models is shown in table 2 supplementary materials, and Fig. 4. In model-1 (clinical model) age (HR 1.07 [1.06–1.08]); CKD (HR 2.26 [1.57–3.27]), HTN (HR 1.37 [1.01–1.87]), male gender (HR 2.08 [95 % CI 1.54–2.82]), smoking history (HR 1.41 [1.01–1.98]), history of HF (HR 1.60 [1.18–2.17]) were independently associated with all-cause mortality. In model-2 (CMR-model) positive stress perfusion (HR 1.55 [1.20–2.01]) and positive LGE (HR 1.32 [1.10–1.58]) were associated with all-cause mortality after correction for the main baseline characteristics, risk factors and history of HF.

**Fig. 5:**
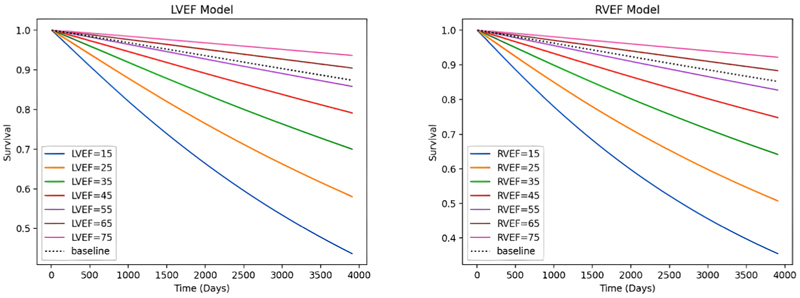
AIF Weibull model for survival incremental analysis for both left ventricular ejection fraction (left) and right ventricular ejection fraction (right). The baseline models (dotted lines) are representation of the average values. LVEF; left ventricular ejection fraction, RVEF; right ventricular ejection fraction.

After testing survival outcome for both ventricular functions, the AFT Weibull model showed reduced survival with lower LVEF and RVEF values, as shown in Fig. 5.

**Fig. 6:**
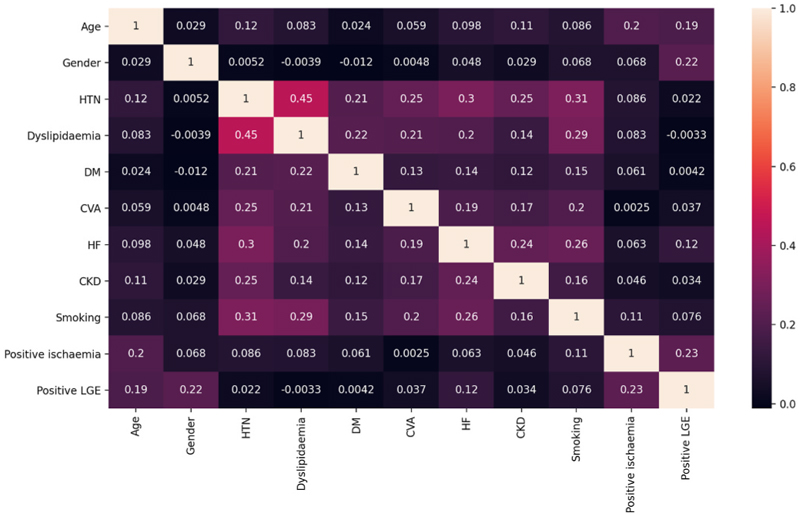
Heatmap showing correlation coefficients for all clinical variables in addition to two CMR variables, the presence of positive stress perfusion and ischaemic scar. All coefficients are displayed in the corresponding cells. CKD; chronic kidney disease, CVA; cerebrovascular accident, DM; diabetes mellitus, HF; heart failure, HTN; hypertension, LGE; late gadolinium enhancement.

After testing for interaction amongst main clinical and CMR variables using heatmap, there was only a moderate correlation between HTN and dyslipidaemia (heatmap correlation coefficient 0.45). Overall, there was no significant interaction between variables (Fig. 6).

Multivariate regression analysis was performed with and without interaction weights, and this showed that the important predictors for ventricular arrhythmia are: HTN (coefficient 0.78, p < 0.001), HF (coefficient 1.38, p < 0.001), positive LGE (coefficient 0.52, p < 0.001), and CVA (coefficient 0.43, p = 0.013).

### 3.5 Machine learning for outcome prediction

After fitting and training machine learning models on clinical variables to predict mortality, support vector machine (SVM) performed best [F1 score = 0.24, AUC = 0.80].

**Fig. 7:**
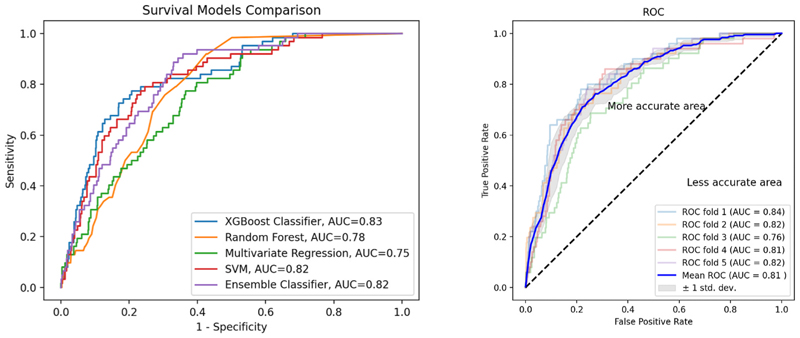
Survival models comparison using area under the curve (AUC). Left is comparison of all models, right is the results of K-fold cross validation of SVM model showing mean AUC of 81 %. SVM; support vector machine.

After the addition of CMR variables, including positive perfusion, positive LGE and LVEF, most machine learning models performed better, however, the improvement was not statistically significant (table 2 supplementary materials). SVM remained the best model with the highest f1 score [F1 score = 0.29, AUC = 0.82]. The difference between AUC of best non-linear model and AUC of the linear regression model was statistically significant (Delong p < 0.001). All results are shown in table 2 supplementary materials. Comparison of all AUCs is shown in Fig. 7.

## 4 Discussion

The main finding of our study were: First, extraction of large unstructured dataset was feasible using data mining techniques and utilising CogStack searching engine and NLP AI algorithms. Second, The extracted data from EHR along with stress perfusion CMR reports on SP-CMR population were analysed in the same pipeline to predict mortality and ventricular arrhythmia. Ventricular function, stress perfusion and LGE results from CMR reports were important predictors of survival. The analysis of important clinical risk factors for outcome prediction were identified and combined with CMR parameters for improved prediction.

Our single large centre study has used a novel data extraction approach. Extraction of the unstructured data used for this project was possible using novel data mining techniques. The use of Cogstack allowed us to manipulate and extract large datasets using NLP models. With our study we showed that such approaches are technically feasible and allow researchers to access a vast amount of data without significant manual efforts. This approach has been used in recent studies [14–16] and the evidence of the feasibility of using automated data mining and AI techniques in health care research is building. A recent systematic review of NLP methods and applications in Cardiology revealed over 37 studies developing and applying NLP in various conditions, such as heart failure, imaging, CAD, electrophysiology, general cardiology and valvular heart disease. Most studies used NLP to identify patients with specific diagnosis and extract disease severity using rule-based NLP methods. Some used NLP algorithms to predict clinical outcomes [17]. A major limitation is the inability to aggregate findings across studies due to vastly different NLP methods, evaluations and reporting.

The data extraction acquired in this project revealed clinically highly plausible results. Our large centre experience shows the significant impact of the findings of positive stress perfusion study or LGE on survival in our adult population. This is in line with previously published data [18]. Despite the side effects of stress agents and the reported cases of abandoned stress perfusion study due to severe adverse effects, the total number of such cases is still considered low (2 %) and the risk of complications is even lower (<1 %). Technical difficulties were found in some cases (2 %), which indicates the challenges in performing a good quality stress perfusion imaging. This requires accurate planning of the three ventricular slices, testing the sequence, testing the equipment to deliver the contrast at the right acquisition time, while communicating clearly with the patient and watching electrocardiography (ECG) signal during the increasing heart rate due to the administration of stress agents. Putting all together, it is crucial to have a well-trained and organised team during all SP-CMR studies with sufficient experience in performing the stress protocol as well as dealing with any adverse events.

Our data confirms the strong predictive value of SP-CMR for all-cause mortality and the incidence of ventricular arrhythmias. Furthermore, it can reduce referrals for unnecessary invasive strategy tests, which could potentially be associated with complications. We found no effect of the localisation of an ischaemic perfusion or myocardial scar on outcome. However, the global scar and ischaemia assessment was a good predictor for survival.

Amongst all clinical risk factors, HF and HTN were strong predictors for both all-cause mortality and ventricular arrhythmias after adjusting for covariates, meanwhile our correlation analysis showed no important interaction between variables. Other clinical risk factors such as age, male gender, smoking history and CKD showed prediction power for all-cause mortality only. The survival prediction from left and right ventricular function showed stepwise decremental survival for lower ejection fraction values, in keeping with the evidence from medical literature [19].

Recently Pezel et al. showed in their large study on 31,752 patients that ML score including clinical and SP-CMR data exhibited a higher prognostic value to predict 10-year death compared with all traditional clinical or CMR scores [20]. In our study the application of non-linear machine learning models to predict outcome has also performed better than linear regression models. Amongst the different non-linear models presented, SVM had the best performance when relying on clinical variables alone, followed by Ensemble Classifier and XGBoost. After the addition of CMR variables (positive perfusion, positive LGE, LVEF), most machine learning predictions have improved and SVM remained the best performing model (AUC 0.82, F1 score 0.29). Non-linear prediction facilitates the learning of more complex relationships in the data, and there are various algorithms, which have been successfully used in different applications. SVMs are a set of supervised learning methods used for classification and regression. They have the advantages of being effective in high dimensional spaces and using a subset of training points in the decision function, which is memory efficient. They are one of the most robust prediction methods, being based on statistical learning frameworks [21].

Overall, an automated pipeline has been created to take unstructured data, pre-process and extract relevant information, pass the information for survival analysis, choose the most relevant predictors, and train various algorithms to vote for the best model with the strongest prediction of outcomes, outperforming conventional statistical analysis, and performed in a short period of time, with more computational resources and very minimal manpower. (graphical abstract).

## 5 Limitations

A significant number of cases were excluded (26 %), this may occur at a higher frequency, especially in centres with less expertise. Excluding studies with less than good quality would be expected to inflate the importance of CMR measures and produces some bias of the results.

The NLP model in this study was not exclusive and some data extraction was performed using CogStack search engine of instances in texts and structured fields. Another limitation is that CMR reports at the time of this study were not included in NLP training and manual extraction was performed, future work will aim to train and be able to provide NLP models capable of extracting all relevant EHR and CMR reports data.

This was a single large centre study and only tested on one population, therefore generalisation should be with caution. Large scale multicentre study is required on much larger dataset to be able to develop more reliable and applicable models, which can be used on a wide range of health care systems.

## 6 Conclusion

AI-based data extraction and analysis tools are becoming more available and bringing new insights into unstructured health data records. Our large single centre experience study shows that this approach is feasible and reveals plausible data. With this we were able to confirm the predictive value of SP-CMR for CAD assessment. Positive ischaemic test and the presence of LGE was associated with a higher risk of mortality regardless of clinical risk factors.

Machine learning models can learn non-linear patterns in health record data and show stronger prediction of outcome compared to conventional linear methods. With the potential for integration into a fully automated pipeline, this approach could provide automated risk assessment in the near future.

## Figures and Tables

**Table 1:** Baseline characteristics by age subgroups.

	Total (n = 4188)	<65 years (n = 2049)	65–75 years (n = 1253)	>75 years (n = 886)	P value for trend
Death	252 (6)	38 (2)	71 (7)	143 (16)	<0.001*
Sex					0.539
Male	2659 (63)	1288 (63)	795 (63)	576 (65)	
Female	1529 (37)	761 (37)	458 (37)	310 (35)	
Clinical risk factors					
Smoking	487 (12)	190 (9)	184 (15)	113 (13)	<0.001*
DM	168 (4)	75 (4)	58 (5)	35 (4)	0.386
HTN	1534 (37)	648 (32)	533 (43)	353 (40)	<0.001*
Dyslipidaemia	824 (20)	338 (16)	317 (25)	169 (19)	<0.001*
CVA	408 (10)	153 (7)	153 (12)	102 (12)	<0.001*
CKD	204 (5)	54 (3)	71 (6)	79 (9)	<0.001*
Previous MI	1014 (24)	475 (23)	333 (26)	206 (23)	0.066
Heart failure	646 (15)	248 (12)	219 (17)	179 (20)	<0.001*
Arrhythmia					
AF	611 (15)	199 (10)	216 (17)	196 (22)	<0.001*
Atrial flutter	166 (4)	63 (3)	67 (5)	36 (4)	0.05*
1st degree HB	140 (3)	39 (2)	46 (4)	55 (6)	<0.001*
2nd degree HB	4 (<1)	1 (<1)	0 (0)	3 (<1)	0.028*
3rd degree HB	39 (1)	14 (<1)	14 (1)	11 (1)	0.252
VT	307 (7)	138 (7)	91 (7)	78 (9)	0.142
VF	53 (1)	28 (1)	16 (1)	9 (1)	0.737
Field strength					0.04*
1.5T	1957 (47)	1010 (49)	578(46)	369 (42)	
3T	2231 (53)	1039 (51)	675 (54)	517 (58)	
Stress agent					0.129
Adenosine	3719 (89)	1828 (89)	1121 (89)	770 (87)	
Regadenosine	469 (11)	221 (11)	132 (11)	116 (13)	
LVEF	55 ± 13	57 ± 12	55 ± 14	51 ± 15	<0.001*
RVEF	58 ± 10	58 ± 9	59 ± 10	58 ± 11	0.011*
+ve ischaemia	1180 (28)	427 (21)	412 (33)	341 (38)	<0.001*
+ve LGE	1275 (30)	424 (21)	428 (34)	423 (48)	<0.001*

Values are presented as number (%) for categorical variables, mean ± standard deviation for continuous variables. AF = atrial fibrillation; CKD = chronic kidney disease; CVA = cerebrovascular accident; DM = diabetes mellitus; HB = heart block; HTN = hypertension; LGE = late gadolinium enhancement; LVEF = left ventricular ejection fraction; MI = myocardial infarction; RVEF = right ventricular ejection fraction; T = tesla; VF = ventricular fibrillation; VT = ventricular tachycardia.
